# Application of chitosan-based drug delivery systems in the treatment of bacterial diseases: a review

**DOI:** 10.1080/10717544.2025.2514140

**Published:** 2025-06-10

**Authors:** Huan Huang, Yaxin Zhou, Jiehang Li, Zhijin Zhang, RongJia Han, Jingru Zuo, Yubin Bai, Jiyu Zhang

**Affiliations:** a Key Laboratory of New Animal Drug Project of Gansu Province, Lanzhou, China; b Ministry of Agriculture, Key Laboratory of Veterinary Pharmaceutical Development, Lanzhou, China; c Lanzhou Institute of Husbandry and Pharmaceutical Sciences, Chinese Academy of Agricultural Sciences, Lanzhou, China

**Keywords:** Chitosan, drug delivery system, bacterial disease

## Abstract

Bacterial diseases are a significant challenge to human and animal health. The current treatment methods still have obvious shortcomings, such as poor targeting, low bioavailability, high side effects and drug resistance. Chitosan, with its outstanding biocompatibility, biodegradability, adhesiveness, antimicrobial properties, and ability to minimize drug side effects while improving bioavailability and therapeutic outcomes, serves as an ideal material for drug delivery systems, presenting a promising strategy for treating bacterial diseases. In this review, we briefly summarize the preparation methods of chitosan-based drug delivery systems and their application in the treatment of bacterial infections. The advantages of preparation of different types of chitosan-based drug delivery systems are discussed, supported by examples demonstrating their ability to improve drug antimicrobial activity, targeting, and bioavailability. Moreover, the current challenges, limitations, and future perspectives in this field were discussed, laying the groundwork for further development of chitosan-based drug delivery systems as high-performance and safe antimicrobial therapeutics.

## 1. Introduction

Bacterial diseases pose a severe threat to human and animal health, leading to significant economic losses in both the medical and veterinary fields (Gupta 2016; Al-Iede et al. 2024); Specifically, And the World Health Organization reports that approximately 700,000 people die each year from bacterial drug-resistant infections, and 10 million people are expected to die each year by 2050. The World Health Organization estimates that by 2050, the global economic cost of drug-resistant infections could be $100 trillion. *S. aureus*, *Escherichia coli* (*E. coli*) and *Salmonella* are common zoonotic pathogens that can cause blood, skin, bowel, soft tissue, and respiratory infections (Ghosh et al. 2023). The bacterial infections are usually accompanied by inflammatory and oxidative reactions. Moreover, *S. aureus*, *E. coli*, *Salmonella* strains are prone to produce biofilms, which enhance drug resistance and seriously threaten the healthy development of the global livestock industry and public health safety. Currently, antibiotics remain the most economical and effective means of preventing and controlling clinical bacterial infectious diseases. They play a crucial role in promoting the healthy development of the breeding industry and protecting the supply of animal-derived food. However, with the increasingly severe problem of bacterial resistance, the research and development speed of antibacterial drugs is much slower than the generation and spread of bacterial resistance, and the clinical antibacterial drug pipeline reserve is seriously insufficient (Zhong et al. 2024). Therefore, there is an urgent need to find new methods to reduce resistance, increase efficacy, and overcome bacterial resistance.

**Figure 1.**
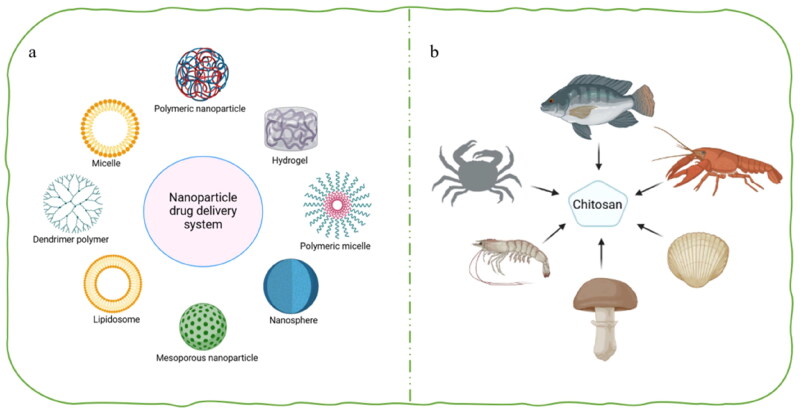
Drug delivery system types and sources of chitosan. (a) Type of drug delivery system. (b) The source of chitosan. (the figure was created with BioRender.com).

Drug delivery systems are of great significance in modern medicine. They can precisely regulate the distribution of drugs in organisms in terms of time, space, and dosage, ensuring that drugs reach the target tissues or organs in the right amount at the right time. This improves the utilization efficiency of drugs, enhances therapeutic effects, reduces costs, and minimizes toxic side effects. In short, drug delivery systems are one of the most effective strategies for tackling bacterial infections. The drug delivery carriers are crucial, encapsulating drugs to improve solubility and bioavailability. With the development of nanotechnology, many nanomaterials have emerged as a new generation of carriers, including hydrogels, liposomes, polymer carriers, micelles, exosomes, vesicles, and dendritic polymers (Figure 1a), which further optimize drug delivery systems and make drug delivery more efficient and targeted.

Chitosan is a natural cationic polysaccharide commonly found in the shells of marine arthropods, such as shrimps, crabs, and fungi (Figure 1b). Compared with other drug carriers such as lipid nanoparticles and polymer micelles, chitosan chitosan has good bioadhesion, biodegradability and inherent antibacterial activity, which has important advantages in drug delivery systems. It also reduces potential toxicity and immune responses, providing a new direction for future drug delivery technologies. Regarding the antibacterial effect of chitosan, this is because amino groups and their cationic properties carried by chitosan can disrupt bacterial cell walls and cell membranes and interfere with the cellular metabolism pathway, giving it antibacterial activity (Helander et al. 2001). In addition, it also can effectively disrupt biofilms, which is its key advantage in the treatment of infections with multidrug resistant bacteria. For example, Zinc-oxO-chitosan nanoparticles showed an inhibition rate of nearly 80% against the biofilm of resistant *E. coli* (Alya et al. 2020); Chitosan could inhibit 80% biofilm formation of MRSA at 2 × MIC, and 4 × MIC was required for *Pseudomonas aeruginosa* (Lu et al. 2025). Chitosan also performs well in drug sustained release. For example, Ailincai (Ailincai et al. 2020) could delay the release of drug-loaded chitosan nanoparticles by 7 days by changing the molar ratio between chitosan and citral. Additionally, chitosan can enhance drug bioavailability by reversibly opening the tight junctions between cells in the stratum corneum of the skin.

However, drug delivery systems have deficiencies such as incomplete and slow drug release, weak biofilm penetration, and poor targeting. With the rapid development of nanotechnology and the deeper insight from biology and other related disciplines, developing of nano-drug delivery systems based on the responsive release of infectious microenvironment (pH, enzymes, reactive oxygen species) offers a promising solution to the limitation of traditional therapeutic drugs in clinical practice. Simultaneously, the structure of chitosan amino, hydroxyl, glucoside bond provides the possibility for the preparation of responsive release nanodelivery system. For example, Asadi (Sepideh et al. 2023) used the property that chitosan has many amine groups, which are protonated and expanded at low pH to release the loaded drug, to build pH-responsive polymer nanocarriers that can release the drug in acidic environments such as tumors and cancers to deliver curcumin. Similarly, glutathione (GSH) response-type nanocarriers were designed based on the functional groups of chitosan, which can accelerate drug release to detect GSH and protect the drug without GSH (Antoniraj et al. 2023). Furthermore, targeted modification of stimulus-responsive drug delivery by adding hyaluronic acid or folic acid (Chandan et al. 2023; Luo et al. 2024) can further improve the stability of the drug delivery system, enhance bioavailability, control drug release, achieve targeted delivery and improve therapeutic effects, thereby improving the therapeutic effects of clinical bacterial diseases.

Chitosan-based drug delivery systems have been widely used, but their effectiveness varies depending on the preparation processes and carriers affecting the efficacy of chitosan-based nanoparticles. Accordingly, this review describes the research progress in using chitosan-based nanoparticles for bacterial diseases from the perspectives of chitosan nanoparticle preparation and preclinical studies. It aimed to provide theoretical references for better clinical applications of chitosan-based drug delivery.

## 2. Preparation and application of chitosan nanoparticles

### 2.1. The preparation of chitosan nanoparticles

Chitosan nanoparticles can be prepared by covalent cross-linking, self-assembly, and ionic cross-linking (Table 1). A highly stable system can be prepared using the covalent cross-linking method, which is based on the reaction of the amino group in chitosan by adding cross-linking agents such as glutaraldehyde, formaldehyde, glyceraldehyde and vanillin (Wang et al. 2024). However, these crosslinkers are toxic and irritating and may affect the biocompatibility of the delivery system and the therapeutic efficacy; therefore, it is necessary to control the dosage strictly. In short, covalent cross-linking method is suitable for applications requiring high stability and strong loading capacity (for example, anti-cancer drugs and tissue engineering scaffolds), but attention should be paid to toxicity issues and operational complexity. The self-assembly method involves forming amphiphilic chitosan blocks or graft copolymers into nanosphere drug-carrying systems using hydrophilic and hydrophobic interactions (Yuan et al. 2024); It is low cost and simple to operate, but the obtained nanoparticles have large particle size and poor physical stability, which are suitable for loading with small molecule drugs, proteins, peptides, gene drugs, etc. (e.g. DNA/siRNA complexes, oral vaccine carriers). It has the effects of increasing drug solubility, improving drug stability, reducing drug toxicity and targeting. Chitosan nanoparticles formed by cross-linking chitosan amino groups with polyanions use the ionic cross-linking method. Its stability is poor, but the operating conditions are mild and simple, and does not involve organic solvents, is the most widely used method. It is especially suitable for loading large molecules such as proteins and peptides, and has the possibility of large-scale production. For example, Owczarek (Monika et al. 2023) used the ion cross-linking method to prepare spherical chitosan nanoparticles. With sodium tripolyphosphate as a common cross-linking agent (Mu et al. 2024). Its concentration and ratio with chitosan specifically influence the particle size, encapsulation rate, and drug loading of nanoparticles (Conte et al. 2023; Essid et al. 2023; Jalal et al. 2023; Kancha et al. 2024; Latifi et al. 2024) (Table 2). An increase in the concentration of chitosan and sodium tripolyphosphate in the system tends to produce nanoparticles with larger particle size (Gutiérrez-Ruíz et al. 2024), but the larger the particle size of nanoparticles, the worse the stability. In addition, the chitosan nanoparticles prepared by ion cross-linking method are unstable. In order to solve this problem, researchers have conducted a series of studies. Wang et al. (2024) formed chitosan nanoparticles by chemical cross-linking by reacting the amino and hydroxyl groups on the molecular chain of chitosan with chemical cross-linking agents. The stable and functional chitosan nanoparticles were obtained by adding anions with the potential to disrupt the *S. aureus* biofilm to the chitosan nanoparticles by ion cross-linking. Furthermore, β-cyclodextrin grafting on chitosan can enhance the function of chitosan nanoparticles to improve the solubility of drugs, promote the absorption of drugs by bacteria, and also provide an idea for reducing drug residues in animal-derived foods (Ding et al. 2018).

**Table 1.** Preparation method of chitosan nanoparticles and its advantages and disadvantages.

Preparation method	Stability	Toxicity	Process complexity	Drug loading type	Apply
Covalent cross-linking	High	Middle- high	High	Hydrophobic/sustained-release drugs	Cancer drugs, implant materials
Self-assembly	Low-middle	Low	Middle	Nucleic acids/hydrophobic molecules	Gene therapy, oral vaccine carrier
Ionic cross-linking	Middle	Low	Low	Hydrophilic/biological macromolecules	Protein vaccine delivery

**Table 2.** Effects of preparation ratio of chitosan nanoparticles on EC and LC.

Chitosan concentration(mg/mL)	TPP concentration (mg/mL)	Ratio of CS to TPP	Size (nm)	Encapsulation efficiency (EC)	Loading capacity (LC)	References
2.8	2	5:2	172 ± 21.00	80.05%	84.26%	(Kancha et al. 2024)
2	1	5:1	46.61 ± 18.16	86.33%	30.17%	(Latifi et al. 2024)
1	1	2:1	480 ± 14.55	92.58%	–	(Essid et al. 2023)
1	0.5	4:1	133 ± 5.00	69.00 ± 13.00%	–	(Jalal et al. 2023)
1	5	1:1	469.31 ± 9.78	30.30 ± 1.91%	–	(Conte et al. 2023)
1	5	10:1	121.22 ± 2.43	76.18 ± 3.16 %	–	(Conte et al. 2023)

### 2.2. Application of chitosan nanoparticles in treating bacterial diseases

**Figure 2.**
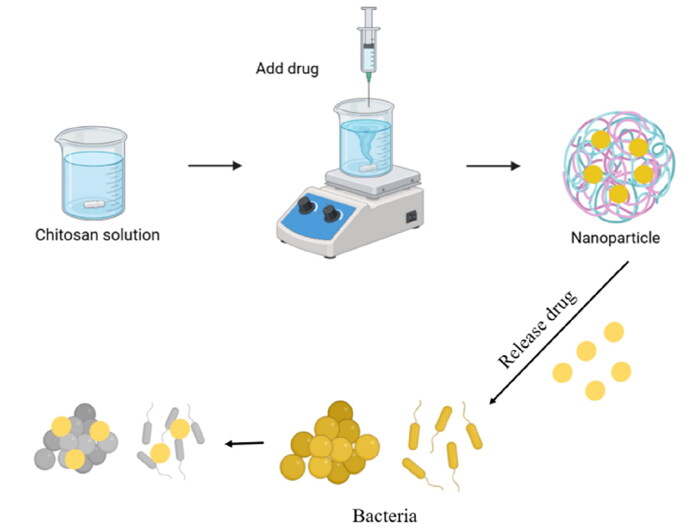
Preparation and antibacterial activity of chitosan nanoparticles. (the figure was created with BioRender.com).

In the aspect of antibacterial, chitosan can enhance the antibacterial ability of drugs. For example, numerous studies have confirmed that drug-loaded chitosan nanoparticles can enhance the inhibition ability of *S. aureus*, *Pseudomonas aeruginosa,* and other bacteria while also reducing inflammation and oxidative damage caused by bacterial infection (Figure 2; Table 3) (Ghazal et al. 2023; Mehra et al. 2024; Wang et al. 2024); Similarly, the antibacterial effect of nano-composite composed of chitosan and zinc oxide on *Helicobacter pylori* is better than that of some conventional antibiotics (Fallahizadeh et al. 2025). And in tobramycin and chitosan or silver nano complex coupling, its inhibition effect on *Pseudomonas aeruginosa* is superior to the simple use of tobramycin (Shahid et al. 2024). In terms of chitosan binding with metals, Asvar et al. (2024) indicated that chitosan works better when combined with multiple metals (silver, copper, zinc) than a single metal. Similarly, chitosan nanoparticles coated with multiple drugs or combined with other therapeutic methods can also overcome the limitations of a single drug, a single method of antibacterial. For example, gold nanoparticles conjugated with antibodies to streptomycin can be synergized with chemical photothermal therapy to achieve a better antibacterial effect (Jiang et al. 2024).

**Table 3.** Application of chitosan nanoparticle delivery system.

Name	Drug	Bacterial	Type of inflammation	Action factor	Deficiency	References
BCNPs	Berberine	Klebsiella pneumonia; Bacillus subtilis	–	–	Chemical crosslinkers have certain toxicity and require special concentration and dosage	(Mehra et al. 2024)
CS/PN NPs	Echinacea pallida	Staphylococcus aureus	–	–	Echinacea pallida is toxic in high concentrations	(Ghazal et al. 2023)
ISL@RL-CS NPs	Isoliquiritigenin	MRSA	–	–	Chemical crosslinkers have certain toxicity and require special concentration and dosage	(Wang et al. 2024)
Cs/AgNCs	Tobramycin	Pseudomonas aeruginosa	–	–	Expensive	(Shahid et al. 2024)
CS-nAg; CS-nCuO; CS-nZnO; CS-nACZ	–	Staphylococcus aureus; Escherichia coli	–	–	potential adverse environmental effects and toxicity	(Asvar et al. 2024)
EUSO-CSNPs	Eucommia ulmoides seed essential oil	Staphylococcus aureus; Escherichia coli	–	–	The drug load is relatively low	(Jiang et al. 2024)
CNMs	Bioactive peptides	–	Escherichia coli infection	TNF-α, NF-κB	–	(Kuang et al. 2021)
ACs/CuONPs	p-aminobenzoic acid	–	Helicobacter pylori infection	COX-2 enzyme	–	(Noura et al. 2023)

A bacterial infection usually causes an inflammatory response, usually accompanied by oxidative damage, further exacerbating the inflammatory response and increasing the treatment difficulty. Studies have shown that chitosan has synergistic anti-inflammatory effect of drugs, such as, the anti-inflammatory activity of curcumin nanoparticles was superior to that of curcumin (Muhammad et al. 2023). In-depth studies have demonstrated that peptide-coupled chitosan nanoparticles can inhibit the inflammation caused by *E. coli* by regulating the expression of TNF-α, IL-1β, IL-6, and other inflammatory factors (Kuang et al. 2021), and the mechanism may be related to the regulation of NF-κB signaling pathway (Zhang et al. 2024). Regarding antioxidant effect, chitosan also has synergistic antioxidant effect. For example, the antioxidant ability of chitosan nanoparticles carrying eucommia seed essential oil is better than that of eucommia seed essential oil or chitosan nanoparticles (Jiang et al. 2024). In order to enhance the effect of chitosan nanoparticles, researchers have carried out the following exploration. Maillard reaction between chitosan and glucose by ultrasound can enhance the antioxidant capacity of chitosan nanoparticles (Supapit et al. 2023). Chitosan can be modified to enhance its antibacterial, anti-inflammatory, and anti-oxidation ability before loading drugs to further improve the therapeutic effect of drug delivery. For example, forming bilayer nanoparticles of chitosan with soybean peptides can enable drugs to exhibit excellent antioxidant capacity by reducing the expression level of malondialdehyde (Li et al. 2024).

Bacterial biofilm formation enhances bacterial resistance to antimicrobial drugs, leading to persistent infection. Thus, it enhances the anti-biofilm ability of drugs or drug carriers. According to statistics, chitosan nanoparticles have different degree of enhance drug eradication of *Pseudomonas aeruginosa*, *E. coli* and *S. aureus* biofilm (Jiaman et al. 2022; Mohammadreza et al. 2023; O’La et al. 2024). An in-depth study concluded that the inhibitory mechanism of chitosan nanoparticles containing epigallocatechin-3-gallate on bacterial biofilm may be related to its influence on bacterial biofilm production by modifying cell morphology (Aragão et al. 2025). To achieve better inhibition of biofilm formation, researchers have explored drugs capable of degrading biofilms and loading them onto chitosan nanoparticles to enhance the biofilm removal effect. For example, modification of chitosan nanoparticles with biofilm-diffusion-active D-tyrosine significantly improved the biofilm elimination ability of chitosan nanoparticles (Tang et al. 2024).

Based on the pH difference between normal tissues, tumors and inflammatory tissues, researchers constructed nanocarriers with pH-responsive functions that exhibit different inhibitory effects on bacteria under different pH conditions (Table 4) (Crisiane et al. 2021). The study revealed that the inhibition of *S. aureus* and *E. coli* was significantly improved when the pH drug loading system was coated with curcumin (Ghaffari et al. 2020). Similarly, the pH-responsive chitosan nanocomposites loaded with ciprofloxacin indicated 4 and 2 times increased antibacterial activity against MRSA and *Pseudomonas aeruginosa* at acidic pH, respectively (Ismail et al. 2024). By targeting the pH of the environment, electrostatically adsorbed charge-convertible drug-loaded nanoparticles can avoid damaging normal cells (Wang et al. 2024). Due to the complex intestinal environment, the low bioavailability of the drug after oral administration results in poor clinical treatment of gastrointestinal diseases, while pH-sensitive chitosan can overcome this limitation (Noura et al. 2023). For example, chitosan nanoparticles loaded with berberine hydrochloride with intestinal targeting can release the drug in a pH = 7.4 environment to treat ulcerative colitis (Linfeng et al. 2022). This is consistent with the Armana (Abdollahy et al. 2024) results, which demonstrated that a load of chitosan nanoparticles can prevent drugs from being released in a strongly acidic environment. While Yu (Yu et al. 2020) research revealed that chitosan nanoparticles modified with octenyl succinic anhydride exhibited good stability at pH = 7.4, the drug-loading system released the drug in large quantities under weakly acidic conditions. This behavior may be related to factors such as the percentage of encapsulation and structural changes in chitosan. In addition to the pH-responsive drug delivery system, the GSH-based reduced-oxidized chitosan nanoparticles indicated significant inhibition of pro-inflammatory factors and efficient and safe delivery (Antoniraj et al. 2023).

**Table 4.** Application of responsive chitosan nanoparticle drug delivery.

Stimulus type	Drug	Bacterial	Type of inflammation	Release condition	References
pH	–	Staphylococcus aureus	–	pH = 5.5	(Crisiane et al. 2021)
pH	Ciprofloxacin	MRSA; Pseudomonas aeruginosa	–	pH = 6.0	(Ismail et al. 2024)
pH	Sodium caseinate	S. mutans bacterial	–	pH = 5.7	(Wang et al. 2024)
pH	Curcumin	Staphylococcus aureus; Escherichia coli	–		(Ghaffari et al. 2020)
pH	Thymol	Botrytis cinerea	–	pH = 4.0/5.5	(Xiaomin et al. 2023)
pH	berberine hydrochloride	ulcerative colitis	–	pH = 7.4	(Linfeng et al. 2022)
pH	5-aminosalicylic acid and hesperidin	ulcerative colitis	–	pH = 7.4	(Abdollahy et al. 2024)
pH	Curcumin and quercetin	–	LPS induced macrophage inflammation	pH = 6.0	(Yu et al. 2020)
redox-responsive	Prednisone	–	airway inflammation in asthma	glutathione	(Antoniraj et al. 2023)
Thermo-Responsive	Hydroxytyrosol	–	ulcerative colitis	37 °C	(Valentino et al. 2022)

In summary, chitosan nanoparticles offer advantages such as simple preparation, improved drug solubility, enhanced drug efficacy, good biocompatibility, and controlled drug release. However, there are some technical challenges in mass production.

## 3. Preparation of chitosan-based hydrogel and applications

### 3.1. Chitosan-based hydrogel preparation

In addition to chitosan nanoparticles, chitosan hydrogels are also an important type of drug delivery system, which has unique properties and applications in the treatment of bacterial diseases. The skin, as the first line of defense to protecting the body’s tissues and organs from harm, is highly susceptible to bacterial infections. When damaged and improperly cared for, this can trigger the body’s immune response, leading to inflammation and tissue damage. Hydrogels, with their highly hydrophilic three-dimensional mesh structure, are particularly effective in precisely controlling drug release and have low rejection rates by biological tissues, making them an ideal material for wound dressing (Sharma and Singh 2024). Hydrogels derived from the extracellular matrix of specific tissues have proven effective in promoting cell proliferation, protecting against arthritis, and tissue regeneration in nude mice with meniscal injuries (Yuan et al. 2017).

**Figure 3.**
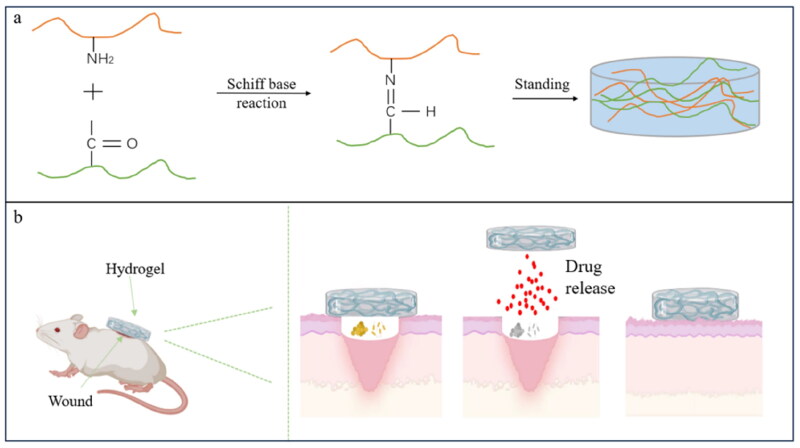
Preparation of hydrogel and its antibacterial effect. (the figure was created with BioRender.com).

Hydrogels can be categorized into physically cross-linked, chemically cross-linked and complex network gels. Physical cross-linking uses polymer hydrophobic, hydrogen bond, van der Waals force, and electrostatic adsorption formation, which is reversible and self-study renaturation of hydrogels. However, hydrogels prepared using this method often exhibit weak mechanical properties, which can be enhanced by the synergistic effect of solvent substitution or adding metal ions. For example, Ou et al. (2024) fabricated a double-network polyacrylamide/calcium alginate/ethanol hydrogel with excellent mechanical properties using free radical polymerization and solvent exchange strategy. Zhang et al. (2024) improved the mechanical properties of chitosan-sodium alginate hydrogel to 2.6 times the original value by adding Ca^2+^. The chemical cross-linking method is characterized by rapid formation, stable structure, and better mechanical strength than the physical cross-linking method. However chemical cross-linking hydrogels tend to be tightly prepared and often require adding toxic chemical reagents, which makes the hydrogel less biocompatible. Xie et al. (2024) indicated that a green and highly swellable chitosan-based hydrogel could be prepared using N-methylene diacrylamide as a crosslinker. Complex network gels are a hybrid system consisting of physical and chemical cross-linking. For example, Peng et al. (2024) prepared safe and long-stable chitosan-based hydrogels using Schiff base reactions between chitosan amino and aldehyde groups and hydrogen bonding reactions. Wang et al. (2024) prepared quaternary ammonium chitosan-based hydrogels with self-healing, slow release and excellent rheological properties, employing both the Schiff base and electrostatic synergistic cross-linking (Figure 3a). Consequently, chitosan-based hydrogels with various functionalities can be prepared by a variety of chemical bonding reactions. Hydrogels with excellent flexibility, adjustable mechanical properties, and biological tissue rejection are ideal carriers for use as functional soft materials.

### 3.2. Application of chitosan hydrogel in treating bacterial disease

Hydrogels have strong adhesion, antimicrobial, and wound healing promoting abilities and can be used to treat wound infections caused by bacteria such as *Pseudomonas aeruginosa* and *S. aureus*. However, chitosan’s macromolecular structure and hydrogen bond limits its application. Therefore, it is necessary to modify the function of chitosan by quaternization, carboxymethylation, and guanidinium to improve the antibacterial ability of chitosan-based hydrogels. The strength of hydrogel adhesion plays a crucial role in preventing wound infection and antimicrobial effects. Chitosan hydrogel modification can significantly improve its adhesion. For example, the adhesion ability of quaternary ammonium chitosan hydrogels is significantly better than commercial antibacterial hydrogels (Andreica et al. 2024); The modified N-succinyl chitosan with negatively charged carboxylate ions can enhance the adhesion properties of the hydrogel (Srimai et al. 2024); The adhesive strength of chitosan biguanide is six times stronger than that of commercial fibrin glue (Shi et al. 2024). In antimicrobial capacity, hydrogels made from borated chitosan derivatives inhibited *S. aureus* and *E. coli* by 50% more effectively than commercial chitosan hydrogels (Liang et al. 2024). However, Yu et al. (2024) noted that chitosan exhibited almost no inhibitory effect on *E. coli*, but chitosan loaded with silver ions can disrupt the integrity of the bacterial biofilm, thereby inhibiting *E. coli*. The high cost of metal ions limited its wide application. Chitosan hydrogels play a very good therapeutic effect on bacterial wound infection. For example, Wang et al. (2024) research shows that chitosan hydrogels/gelatin inhibition effect of *E. coli* and *S. aureus* reached more than 90%, and the wounds in the chitosan/gelatin hydrogel group indicated a natural color when the wounds in the untreated group remained dark red (Figure 3b); For back wound infection caused by *Pseudomonas aeruginosa* in rats, the wound closure rate of chitosan-based hydrogel was about 92% (Kolarijani et al. 2024); Similarly, chitosan hydrogels showed significant antibacterial and anti-biofilm activity against multidrug-resistant *Pseudomonas aeruginosa* when applied to pig skin wound infection (Moon et al. 2023). Further studies show that chitosan hydrogels also play an important role in promoting wound healing. For example, Shi (Shi et al. 2024) revealed that hydrogels composed of chitosan bisguanide, oxidized dextran, and tannins exhibited excellent antimicrobial efficacy and wound-healing-promoting ability and were superior to commercial fibrin glue. This effectiveness likely stems from the hydrogel’s ability to eliminate bacteria, reduce inflammation reactions, and promote angiogenesis in wound healing and regeneration of bacterial infection (Tan et al. 2024).

Compared with traditional hydrogels, the modified hydrogels have more advantages such as pH response and targeted release. For example, carboxymethyl-modified pH-responsive chitosan hydrogels can release drugs in alkaline environments, exhibit excellent bacteriostatic effects, and demonstrate antioxidant activities with scavenging capacities of greater than 58% for 2,2-diphenyl-1-picrylhydrazyl (DPPH) radicals and hydroxyl radicals (Xiong et al. 2024). Additionally, the DPPH radical scavenging rate of keratin/sodium and alginate/carboxymethyl chitosan modified by tannic acid could reach over 95% (Zhu et al. 2024). To achieve targeted drug release, Wang et al. (2024) made use of the electrostatic interaction between positive and negative ions to prepare the lactoferrin-chitosan complex hydrogel released in the small intestine, which greatly improved the efficacy of the drug.

In summary, chitosan-based hydrogels play a significant role in treating bacterial-induced wound infections. However, *E. coli* and *S. aureus* are prone to biofilm formation, which helps them evade drugs, and there are few reports on the anti-biofilm effect of chitosan-based hydrogels. To address this report, these chitosan-based hydrogels can be modified by drugs with the dispersing biofilm function, such as D-tyrosine, or drugs with antibacterial and anti-biofilm properties can be explored.

## 4. Chitosan liposome preparation and application

### 4.1. Chitosan-based liposome preparation

At present, liposome-nanoparticle drug delivery system is one of the most promising drug carriers. Liposomes are formed by phospholipid bilayer bag similar to the biological membrane structure of amphiphilic nanoparticles, which exhibit good biocompatibility, high security, and strong ability drug characteristics (Bozzuto and Molinari 2015). Liposomes are often prepared by thin film dispersion, pH gradient, freeze-thaw, and ethanol injection (Table 5). Hydration phospholipids, drug loading, and particle size control are the key steps in preparing liposomes.

**Table 5.** Preparation method of chitosan-based liposome and its advantages and disadvantages.

Method	Encapsulation efficiency	Particle size control	Process complexity	Applicable drug type	Scale potential	Apply
Thin film dispersion	Low-middle	Poor (post-processing required)	Low	Fat-soluble drugs	High	Laboratory basic research
pH gradient	High	Medium	Middle-high	Weak acid/base drugs	High	Clinical drug delivery (e.g. cancer drugs, antibiotics)
Freeze-thaw	Middle-high	Unevenness	Middle	Macromolecules/sensitive drugs	Low	Biomacromolecules (e.g. proteins, peptides, mRNA encapsulation, gene therapy (DNA), cryoprotectant screening (glycerol)
Ethanol injection	Middle	Best (nanoscale)	Low	Water/fat soluble drugs	Middle	Nanoscale liposomes (e.g. targeted siRNA delivery)

**Figure 4.**
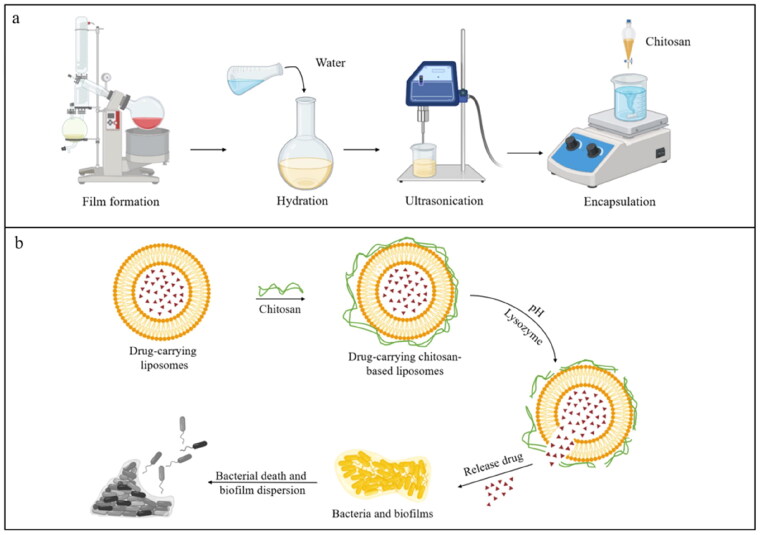
Antibacterial effect of chitosan-based liposomes (the figure was created with BioRender.com).

The thin film dispersion method involves reacting phospholipids and cholesterol in a water bath until a thin film forms. Water is then added for hydration, and it finally obtains uniform and stable nano-liposomes by ultrasonic fragmentation (Shnaikat et al. 2024) (Figure 4a). However, this method is complicated, and the hydration rate is closely related to the drug encapsulation efficiency. For smaller particle sizes, ultrasound and high-pressure homogenization may be required. As the earliest and most basic liposome preparation method, thin film dispersion method has low equipment requirements and is suitable for small batch production of laboratory scale. It can lay the foundation for other liposome preparation methods (such as pH gradient method), but it is not suitable for water-soluble drug encapsulation, and the encapsulation rate of fat-soluble drugs can reach 100%. The pH gradient method offers the advantages of rapid preparation, prevents using organic solvents, high encapsulation efficiency, and good stability. It works by creating a pH difference between the interior and exterior of the liposome, achieved by rapidly adjusting the pH of the aqueous solution containing phosphatidic acid. This method is suitable for applications requiring high encapsulation efficiency and uniform particle size (e.g. delivery of anti-cancer drugs such as doxorubicin, delivery of antibiotics such as amphotericin B, and use as long-acting injections). However, the operation is complex, requires precise control of pH gradient conditions and is costly. This allows the drug to penetrate the membrane in different dissociation states (Large et al. 2021). The freeze-thaw method is similar to the thin-film dispersion method in that phospholipids and cholesterol are first formed by rotating evaporation into thin films and then rehydrated. On the basis of the film dispersion method, freeze-thaw method adds the step of rapid freezing and water bath melting, which can enhance the permeability of the film. However, repeated freezing and thawing will cause damage to heat-sensitive drugs, and the stability of the liposomes prepared is poor. Therefore, the method is suitable for long-term storage or mass production of liposomes (such as vaccines). The ethanol injection method uses an organic solvent to fully dissolve the phospholipids and other substances, which are then slowly injected into an aqueous solution at the appropriate temperature. Finally, the organic solvent is removed to obtain the liposomes. This method is suitable for large-scale industrial production and is fast, safe and reproducible, but the resulting liposomes have heterogeneous particle sizes, weak stability, and difficulty in removing organic solvents, which are prone to potential residues (Vélez et al. 2017). It can be used in nano targeted delivery systems, cosmetic active ingredients (such as vitamin C, astaxanthin, etc.).

### 4.2. Application of chitosan-based liposomes in treating bacterial diseases

As a mature drug delivery vehicle, liposomes have been proven to deliver antibacterial drugs to treat bacterial infections. Liposomes modified with chitosan can exhibit significant bacterial inhibition, such as loading liposomes into a polyvinyl alcohol-chitosan matrix, which can significantly inhibited *E. coli* and *S. aureus* (Shaobing et al. 2022). Compared with the free drug, the liposome chitosan package encapsulates drugs after inhibition of *E. coli* increased 13 times, and inhibition of *E. coli* biofilm can reach 80% (de Souza et al. 2024). It has been reported that N-succinyl chitosan not only protects the liposomes from the attack of gastric acid, but also has the ability of pH responsive release (Figure 4b), In addition, chitosan itself has certain antibacterial ability, so the antimicrobial efficacy of chitosan-encapsulated liposomes against *S. aureus* and *E. coli* was superior to that of single liposomes. Liu (Aiyang et al. 2023) revealed that N-succinyl chitosan-coated liposomes were more effective against *E. coli* due to the thinner cell walls of Gram-negative bacteria. For lung infections caused by Mycobacterium abscessus, chitosan-modified liposomes can be nebulized, and the effect is remarkable (Jacopo et al. 2023). Enzymes can act as the switches that regulate the on-demand release of nanocarriers; therefore, the enzyme-containing drug delivery system can prevent bacterial infection accurately and quickly (Figure 4b). For example, chitosan-based liposomes containing lysozyme could increase the inhibitory ability against *S. aureus* from 72.46% to 100%, and the ability to remove *S. aureus* biofilm was increased to 72% (Dong et al. 2024). In vaccines, chitosan-based liposomes can also be used as an adjuvant system for *Salmonella typhi* pore proteins to enhance immune responses to subunit vaccines (Selin et al. 2023).

In summary, chitosan-based liposome coating can significantly improve drug efficacy and precision delivery ability of drugs.

## 5. Other drug delivery systems based on chitosan

### 5.1. Preparation of chitosan-based micelles, vesicles, microspheres, and exosomes

Chitosan has numerous applications in the micelle, vesicles, microspheres and exosomes. Focusing on the preparation of polymeric micelles, several methods are commonly used, including dialysis, thin-film dispersion method, freeze-drying, and directly dissolved method. The dialysis method is the most commonly used in the laboratory. This method involves the organic solvent being gradually exchanged with water, and the hydrophobic chain segments of the copolymer are gradually coalesced under the influence of the solvent, thus forming a micelle core. The thin film dispersion method involves rotary evaporation to form a thin film, then adding an aqueous solution for hydration and stirring to obtain polymer micelles. The organic solvents in dialysis and film dispersion methods are difficult to remove and must be completely removed by dialysis and freeze-drying (Yuan et al. 2024). The freeze-drying method dissolve the drug and copolymer in a mixed organic solvent and water solution. Then, the freeze-dried powder can be assembled into micelles in the aqueous medium. This method is simple, economical, and prevents organic solvent residues. Although the direct dissolution method is simple, the resulting micelle prepared tends to have weak stability. In conclusion, the film dispersion and freeze-drying methods are not only simple and economical but also suitable for large-scale production.

For the preparation of chitosan vesicles, the common methods include laminar assembly, emulsification, sol-gel and so on. Taking the layered assembly method as an example, the principle is to form multi-layer nanovesicles by alternately depositing chitosan and other polymers, which is highly controllable and versatile (Davidson et al. 2023). For chitosan microspheres, the common preparation methods include emulsion crosslinking, solvent evaporation, spray drying, etc. These methods have their own characteristics, such as emulsion crosslinking method is simple but needs to reduce the concentration of chitosan to avoid adhesion, solvent evaporation method is used to form W/O emulsion by ultrasonic treatment, and spray drying method is suitable for large-scale production (Spogli et al. 2024; Yu et al. 2024; Zhang et al. 2024). Chitosan exosome is a mixture of chitosan solution and exosome, which is formed by physical and chemical methods and purified to obtain a drug carrier system with both biocompatibility and stability (Jabeen et al. 2024).

### 5.2. Application of chitosan micelles, vesicles, microspheres, and exosomes in treating bacterial disease

**Figure 5.**
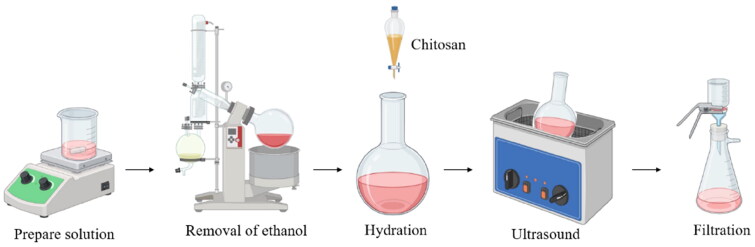
Chitosan-based micelle preparation process. (the figure was created with BioRender.com).

Polymer micelles are core-shell structures formed by the spontaneous assembly of amphiphilic block copolymers in an aqueous solution, which can take on the ball, rod, and capsule alveolar morphology. The hydrophobic environment of the micelle core plays a vital role in insoluble drugs’ solubilization and drug loading rate (Gallego-Arranz et al. 2020). The different ratios and particle sizes of the polymers determine the drug release rate (Kabanov et al. 2002; Akrami-Hasan-Kohal et al. 2024), with full release achieved under optimal conditions (Li et al. 2023). Polymeric micelles offer advantages in improving oral absorption, drug loading, intestinal bioadhesive, and stability of drugs. For example, Zhang et al. (2025) prepared a chitosan-stearic acid micellar delivery system for enhanced oral utilization of paclitaxel using the solvent evaporation-water method (Figure 5). In terms of antibacterial activity, chitosan-based micelles can cooperate with drugs to play an antibacterial role, such as, caffeic acid grafted chitosan self-assembled micelles could protect quercetin, slow down its degradation and enhance its antibacterial activity at low pH (Ren et al. 2023). Chitosan-based micelle of *E. coli* and *S. aureus* inhibitory effect is better than chitosan (Qi et al. 2023) due to the enhanced antimicrobial effect of the micelles by disrupting or forming the cell membrane on the cell surface. These micelles also effectively scavenge biofilm formed by *S. aureus* (Ch et al. 2023).

Vesicles are considered one of the most promising nanocarriers for targeted drug delivery (Miao et al. 2024) because of their long storage period, relative nontoxicity, and high modifability compared with general drug carriers (Mondal et al. 2023). For example, multi-functional nanoscale chitosan vesicles loaded with manganese indicated enhanced antioxidant activity and resistance to *E. coli* over time while maintaining high biocompatibility (Davidson et al. 2023). The antibacterial effect of chitosan-modified vesicles against *S. aureus* was equivalent to that of antibiotics and superior to hyaluronic acid modification (Sally et al. 2023). Meanwhile, chitosan-modified vesicles can increase drug permeability. For example, in bacterial conjunctivitis, chitosan-based vesicles are 1.79 times more permeable than commercial eye drops and prolong drug residence time in the cornea (Alruwaili et al. 2020).

Chitosan microspheres also play a significant role in antibacterial activity. For example, Chitosan microspheres exhibit synergistic drug effects against *S. aureus* and *E. coli* (Spogli et al. 2024) and have excellent stability, long-lasting antibacterial ability, and pH-responsiveness (Yu et al. 2024). Chitosan modified MOSe_2_ nanospheres can kill *Helicobacter pylori* in mice, and have no adverse effect on the homeostasis of intestinal flora. *In vitro* studies showed that the nanospheres also had a good therapeutic effect on helicobacter pylori induced gastritis (Zhang et al. 2024). Exosomes actively secreted by cells experience the advantages of low immunogenicity, stable physicochemical properties, strong tissue penetration ability, and reduced off-target effects (Izzati et al. 2023; Jabeen et al. 2024; Ray et al. 2024). Incorporation chitosan can improve the ability of exosomes against *S. aureus* and *E. coli* (Shi et al. 2024).

In summary, the strong stability and permeability of chitosan-based micelles, vesicles, microspheres, and exosomes provided significant potential and application prospects in treating bacterial diseases.

## 6. Conclusions and future perspectives

Bacterial diseases have seriously threatened human and animal health, creating significant challenges in the medical and veterinary fields. The rapid development of the nanoparticle delivery system broke the limitations of conventional drug treatment, enhancing the bioavailability of antibacterial drugs and targeted release ability, thereby improving the clinical effect and reducing the potential for adverse reactions. As an excellent drug delivery carrier, the source, degree of deacetylation, molecular weight, concentration, and proportion of chitosan in the system exhibit a certain impact on the particle size, drug loading, drug release ability, and targeting ability of chitosan-based drug delivery system. Researchers have addressed the limitations of chitosan through its derivatives or different forms of modification and improved performance. Adding hyaluronic acid, folic acid, and other modifications makes the drug delivery system more accurate to target the lesion site and reduce the side effects of drugs. Meanwhile, nano-delivery systems based on responsive release triggered by changes in the microenvironment (pH, enzymes, and temperature) at the lesion site have promising applications in treating bacterial diseases.

Currently, the research on nano-delivery systems for treating bacterial diseases remains in its infancy and faces many challenges. First, chitosan has excellent antibacterial, anti-inflammatory, and antioxidant effects, and it is necessary to explore and utilize the potential pharmacological effects of chitosan. For example, photodynamic therapy and other methods are combined, thus exerting a more substantial therapeutic effect. Second, the mechanism of the chitosan-based drug delivery system remains unclear, and the target point of action is not precise enough. Moreover, studies on chitosan-based drug delivery systems are limited to *in vitro* and mouse models; therefore, mor experimental data are needed to support their clinical applications. Finally, there are various technical challenges to overcome if mass production is to be realized. In conclusion, chitosan-based drug delivery systems have indicated excellent performance and potential in various fields. In the future, more advanced nano-drug delivery systems can be explored to accurately respond to the microenvironment changes in the organism, accurately target the diseased tissue or even cells, and be promoted to the clinic to realize the production of chitosan-based drug delivery systems.

## Data Availability

Data sharing is not applicable to this article as no new data were created or analyzed in this study.

## References

[CIT0001] Abdollahy A, Salehi M, Mahami S, Bernkop-Schnürch A, Vahedi H, Gharravi AM, Mehrabi M. 2024. Therapeutic effect of 5-ASA and hesperidin-loaded chitosan/Eudragit^®^ S100 nanoparticles as a pH-sensitive carrier for local targeted drug delivery in a rat model of ulcerative colitis. Int J Pharm. 652:123838. doi:10.1016/j.ijpharm.2024.123838. 38266937

[CIT0002] Ailincai D, Porzio W, Marin L. 2020. Hydrogels based on imino-chitosan amphiphiles as a matrix for drug delivery systems. Polymers. 12(11):2687. doi:10.3390/polym12112687. 33202586 PMC7696980

[CIT0003] Aiyang L, Xiuhang C, Shuang Z, et al. 2023. Effects of N-succinyl-chitosan coating on properties of astaxanthin-loaded PEG-liposomes: environmental stability, antioxidant/antibacterial activities, and in vitro release. Int J Biol Macromol. 2023:244. 10.1016/j.ijbiomac.2023.125311 37302627

[CIT0004] Akrami-Hasan-Kohal M, Chouchou A, Blanquer S, Sharkawi T. 2024. Everolimus-encapsulation in Pluronic P123 self-assembled micelles as drug delivery systems for drug-coated balloons. Int J Pharm X. 7:100230. doi:10.1016/j.ijpx.2024.100230. 39668884 PMC11636783

[CIT0005] Al-Iede M, Ayyad DM, Etoom RA, Aldameiry RH, Toubasi AA. 2024. The prevalence and risk factors of methicillin-resistant Staphylococcus aureus among pediatric populations: a systematic review and meta-analysis. Eur J Pediatr. 183(9):3679–3687. doi:10.1007/s00431-024-05672-7. 38970703

[CIT0006] Alruwaili NK, Imam SS, Alotaibi NH, Alhakamy NA, Alharbi KS, Alshehri S, Afzal M, Alenezi SK, Bukhari SNA, Ameeduzzafar, et al. 2020. Formulation of chitosan polymeric vesicles of ciprofloxacin for ocular delivery: box-behnken optimization, in vitro characterization, HET-CAM irritation, and antimicrobial assessment. AAPS PharmSciTech. 21(5), 167. doi:10.1208/s12249-020-01699-9. 32504176

[CIT0007] Alya L, BPS, Mausam M, et al. 2020. A streamlined study on chitosan-Zinc oxide nanomicelle properties to mitigate a drug-resistant biofilm protection mechanism. Front Nanotechnol. 2. 13(21), 1625-1637. doi:10.2174/1389557521666210105122841.

[CIT0008] Andreica B-I, Mititelu-Tartau L, Rosca I, Pelin IM, Nicol E, Marin L. 2024. Biocompatible hydrogels based on quaternary ammonium salts of chitosan with high antimicrobial activity as biocidal agents for disinfection. Carbohydr Polym. 342:122389. doi:10.1016/j.carbpol.2024.122389. 39048229

[CIT0009] Antoniraj MG, Mohankumar R, Kumari HLJ, et al. 2023. Fabrication of chitosan-based redox-responsive polymeric nanoparticles: *in-vitro* and *in-vivo* evaluation for treating airway inflammation in asthma. J Drug Delivery Sci Technol. 84:104473. doi:10.1016/j.jddst.2023.104473.

[CIT0010] Aragão MGB, Tedesco AC, Borges HS, Aires CP, Corona SAM. 2025. Chitosan nanoparticles loaded with epigallocatechin-3-gallate: synthesis, characterisation, and effects against Streptococcus mutans biofilmEpigallocatechin-loaded chitosan nanoparticles: effects against Streptococcus mutans biofilm. Nat Prod Res. 39(9):2550–2557. doi:10.1080/14786419.2024.2302321. 38206896

[CIT0011] Asvar Z, Pirbonyeh N, Emami A, Hashemi S-S, Fadaie M, Ebrahiminezhad A, Mirzaei E. 2024. Enhancing antibacterial activity against multi-drug resistant wound bacteria: incorporating multiple nanoparticles into chitosan-based nanofibrous dressings for effective wound regeneration. J Drug Delivery Sci Technol. 95:105542. doi:10.1016/j.jddst.2024.105542.

[CIT0012] Bozzuto G, Molinari A. 2015. Liposomes as nanomedical devices. Int J Nanomedicine. 10:975–999. doi:10.2147/IJN.S68861. 25678787 PMC4324542

[CIT0013] Ch S, Padaga SG, Ghosh B, Roy S, Biswas S. 2023. Chitosan-poly(lactide-co-glycolide)/poloxamer mixed micelles as a mucoadhesive thermo-responsive moxifloxacin eye drop to improve treatment efficacy in bacterial keratitis. Carbohydr Polym. 312:120822. doi:10.1016/j.carbpol.2023.120822. 37059521

[CIT0014] Chandan G, Pinky S, Shashikant V, et al. 2023. A novel thermoresponsive nano carrier matrix of hyaluronic acid, methotrexate and chitosan to target the cluster of differentiation 44 receptors in tumors. Int J Biol Macromol. :243:125238. doi: 10.1016/j.ijbiomac.2023.125238. 37290545

[CIT0015] Conte R, De Luca I, Valentino A, Cerruti P, Pedram P, Cabrera-Barjas G, Moeini A, Calarco A. 2023. Hyaluronic acid hydrogel containing resveratrol-loaded chitosan nanoparticles as an adjuvant in atopic dermatitis treatment. J Funct Biomater. 14(2):82. doi:10.3390/jfb14020082. 36826881 PMC9959248

[CIT0016] Crisiane AM, Murilo, ÁVR, et al. 2021. The effects of ionic strength and pH on antibacterial activity of hybrid biosurfactant-biopolymer nanoparticles. J Appl Polym Sci. 139(1):51437. doi:10.1002/APP.51437.

[CIT0017] Davidson E, Pereira J, Gan Giannelli G, Murphy Z, Anagnostopoulos V, Santra S. 2023. Multi-functional chitosan nanovesicles loaded with bioactive manganese for potential wound healing applications. Molecules. 28(16):6098. doi:10.3390/molecules28166098. 37630350 PMC10459768

[CIT0018] de Souza JB, de Lacerda Coriolano D, Dos Santos Silva RC, da Costa Júnior SD, de Almeida Campos LA, Cavalcanti IDL, Lira Nogueira MCdB, Pereira VRA, Brelaz-de-Castro MCA, Cavalcanti IMF, et al. 2024. Ceftazidime and usnic acid encapsulated in chitosan-coated liposomes for oral administration against colorectal cancer-inducing Escherichia coli. Pharmaceuticals. 17(6):802. doi:10.3390/ph17060802. 38931469 PMC11206294

[CIT0019] Ding W-Y, Zheng S-D, Qin Y, Yu F, Bai J-W, Cui W-Q, Yu T, Chen X-R, Bello-Onaghise G, Li Y-H, et al. 2018. Chitosan grafted with β-Cyclodextrin: synthesis, characterization, antimicrobial activity, and role as absorbefacient and solubilizer. Front Chem. 6:657. doi:10.3389/fchem.2018.00657. 30687698 PMC6335354

[CIT0020] Dong Y, Wu T, Jiang T, Zhu W, Chen L, Cao Y, Xiao Y, Peng Y, Wang L, Yu X, et al. 2024. Chitosan-coated liposome with lysozyme-responsive properties for on-demand release of levofloxacin. Int J Biol Macromol. 269(Pt 2):132271. doi:10.1016/j.ijbiomac.2024.132271. 38734330

[CIT0021] Essid R, Ayed A, Djebali K, Saad H, Srasra M, Othmani Y, Fares N, Jallouli S, Abid I, Alothman MR, et al. 2023. Anti-candida and anti-leishmanial activities of encapsulated cinnamomum verum Essential oil in chitosan nanoparticles. Molecules. 28(15):5681. doi:10.3390/molecules28155681. 37570651 PMC10419485

[CIT0022] Fallahizadeh S, Yousefi M, Ghasemi A, Sadat SA, Mohtashemi M, Rezagholizade-Shirvan A, Naghmachi M. 2025. Antibacterial and biofilm inhibition of Helicobacter pylori using green synthesized MWCNTs/ZnO/Chitosan nanocomposites. Environmental Technol Innovat. 38:104068. doi:10.1016/j.eti.2025.104068.

[CIT0023] Gallego-Arranz T, Pérez-Cantero A, Torrado-Salmerón C, Guarnizo-Herrero V, Capilla J, Torrado-Durán S. 2020. Improvement of the pharmacokinetic/pharmacodynamic relationship in the treatment of invasive aspergillosis with voriconazole. Reduced drug toxicity through novel rapid release formulations. Colloids Surf B Biointerfaces. 193:111119. doi:10.1016/j.colsurfb.2020.111119. 32464356

[CIT0024] Ghaffari S-B, Sarrafzadeh M-H, Salami M, Khorramizadeh MR. 2020. A pH-sensitive delivery system based on N-succinyl chitosan-ZnO nanoparticles for improving antibacterial and anticancer activities of curcumin. Int J Biol Macromol. 151:428–440. doi:10.1016/j.ijbiomac.2020.02.141. 32068061

[CIT0025] Ghazal G, Hussein NR, Cosme PRD, et al. 2023. Chitosan/pectin nanoparticles encapsulated with echinacea pallida: a focus on antibacterial and antibiofilm activity against multidrug-resistant Staphylococcus aureus. Appl Biochem Biotechnol. 196(10):1-1. doi:10.1007/S12010-023-04709-1. 37656354

[CIT0026] Ghosh C, Das MC, Acharjee S, Bhattacharjee S, Sandhu P, Kumari M, Bhowmik J, Ghosh R, Banerjee B, De UC, et al. 2023. Combating Staphylococcus aureus biofilm formation: the inhibitory potential of tormentic acid and 23-hydroxycorosolic acid. Arch Microbiol. 206(1):25. doi:10.1007/s00203-023-03762-y. 38108905

[CIT0027] Gupta A. 2016. Bacterial diseases of livestock animals and their ompact on human health. Innovare J Sci. 5(1):8–11.

[CIT0028] Gutiérrez-Ruíz SC, Cortes H, González-Torres M, Almarhoon ZM, Gürer ES, Sharifi-Rad J, Leyva-Gómez G. 2024. Optimize the parameters for the synthesis by the ionic gelation technique, purification, and freeze-drying of chitosan-sodium tripolyphosphate nanoparticles for biomedical purposes. J Biol Eng. 18(1):12. doi:10.1186/s13036-024-00403-w. 38273413 PMC10811841

[CIT0029] Helander IM, Nurmiaho-Lassila EL, Ahvenainen R, Rhoades J, Roller S. 2001. Chitosan disrupts the barrier properties of the outer membrane of Gram-negative bacteria. Int J Food Microbiol. 71(2-3):235–244. doi:10.1016/s0168-1605(01)00609-2. 11789941

[CIT0030] Ismail EA, Omolo CA, Gafar MA, Khan R, Nyandoro VO, Salifu EY, Govender T. 2024. Multi-functional pH-responsive and biomimetic chitosan-based nanoplexes for targeted delivery of ciprofloxacin against bacterial sepsis. Int J Biol Macromol. 262(Pt 1):130046. doi:10.1016/j.ijbiomac.2024.130046. 38336334

[CIT0031] Izzati H, Hyeok PD, Yu CM, et al. 2023. Nanomaterials-based exosomes for the diagnostics and drug deliveries of central nervous system diseases. Biochip J. 17(3):293–307. doi:10.1007/S13206-023-00112-4.

[CIT0032] Jabeen F, Zubair IM, Yuguang L, et al. 2024. Vitis vinifera Kyoho-derived exosome-like nanoparticles-based drug delivery and therapeutic modalities for breast cancer therapy. J Drug Delivery Sci Technol. *2024*:92.

[CIT0033] Jacopo F, Nadia HP, Noemi P, et al. 2023. Mucoadhesive rifampicin-liposomes for the treatment of pulmonary infection by mycobacterium abscessus: chitosan or ε-poly-L-lysine decoration. Biomolecules. 13(6). doi:10.3390/BIOM13060924. PMC10296137 37371504

[CIT0034] Jalal RR, Ways TMM, Abu Elella MH, Hassan DA, Khutoryanskiy VV. 2023. Preparation of mucoadhesive methacrylated chitosan nanoparticles for delivery of ciprofloxacin. Int J Biol Macromol. 242(Pt 4):124980. doi:10.1016/j.ijbiomac.2023.124980. 37236558

[CIT0035] Jiaman X, Quan L, Maokun S, et al. 2022. Antibiofilm effect of cinnamaldehyde-chitosan nanoparticles against the biofilm of Staphylococcus aureus. Antibiotics. 11(10):1403. doi: 10.3390/ANTIBIOTICS11101403. PMC9598764 36290061

[CIT0036] Jiang X, Yu Y, Ma S, Li L, Yu M, Han M, Yuan Z, Zhang J. 2024. Chitosan nanoparticles loaded with Eucommia ulmoides seed essential oil: preparation, characterization, antioxidant and antibacterial properties. Int J Biol Macromol. 257(Pt 2):128820. doi:10.1016/j.ijbiomac.2023.128820. 38103671

[CIT0037] Kabanov AV, Batrakova EV, Alakhov VY. 2002. Pluronic block copolymers as novel polymer therapeutics for drug and gene delivery. J Control Release. 82(2-3):189–212. doi:10.1016/s0168-3659(02)00009-3. 12175737

[CIT0038] Kancha MM, Alizadeh M, Mehrabi M. 2024. Comparison of the protective effects of CS/TPP and CS/HPMCP nanoparticles containing berberine in ethanol-induced hepatotoxicity in rat. BMC Complement Med Ther. 24(1):39. doi:10.1186/s12906-023-04318-9. 38225618 PMC10789080

[CIT0039] Kolarijani NR, Mirzaii M, Zamani S, Maghsoodifar H, Naeiji M, Douki SAHS, Salehi M, Fazli M. 2024. Assessment of the ability of Pseudomonas aeruginosa and Staphylococcus aureus to create biofilms during wound healing in a rat model treated with carboxymethyl cellulose/carboxymethyl chitosan hydrogel containing EDTA. Int Wound J. 21(5):e14878. doi:10.1111/iwj.14878. 38682897 PMC11057379

[CIT0040] Kuang M, Yu H, Qiao S, Huang T, Zhang J, Sun M, Shi X, Chen H. 2021. A novel nano-antimicrobial polymer engineered with chitosan nanoparticles and bioactive peptides as promising food biopreservative effective against foodborne pathogen E. coli O157-caused epithelial barrier dysfunction and inflammatory responses. IJMS. 22(24):13580. doi:10.3390/ijms222413580. 34948377 PMC8706205

[CIT0041] Large DE, Abdelmessih RG, Fink EA, Auguste DT. 2021. Liposome composition in drug delivery design, synthesis, characterization, and clinical application. Adv Drug Deliv Rev. 176:113851. doi:10.1016/j.addr.2021.113851. 34224787

[CIT0042] Latifi A, Esmaeili F, Mohebali M, Yasami-Khiabani S, Rezaeian M, Soleimani M, Kazemirad E, Amani A. 2024. Chitosan nanoparticles improve the effectivity of miltefosine against Acanthamoeba. PLoS Negl Trop Dis. 18(3):e0011976. doi:10.1371/journal.pntd.0011976. 38527059 PMC10962830

[CIT0043] Li P, Liu Q, Xiang Z, Wang J, Wu W-X, Yi W-J. 2023. ROS-responsive core crosslinked micelles by combination of enzymatic polymerization and thiol-ene click chemistry for anticancer drug delivery. Eur Polym J. 199:112473. doi:10.1016/j.eurpolymj.2023.112473.

[CIT0044] Li S, Guan T, Lv H, Cai Y, Cao W, Zhang Z, Song H, Cao H, Guan X. 2024. Fabrication of diosmin loaded food-grade bilayer nanoparticles with modified chitosan and soy peptides and antioxidant properties examination. Food Chem X. 21:101237. doi:10.1016/j.fochx.2024.101237. 38426075 PMC10902142

[CIT0045] Liang S, Chen H, Chen Y, Ali A, Yao S. 2024. Multi-dynamic-bond cross-linked antibacterial and adhesive hydrogel based on boronated chitosan derivative and loaded with peptides from Periplaneta americana with on-demand removability. Int J Biol Macromol. 273(Pt 2):133094. doi:10.1016/j.ijbiomac.2024.133094. 38878926

[CIT0046] Linfeng S, Xiangjiang N, Wenjie L, et al. 2022. Mucus-penetrating alginate-chitosan nanoparticles loaded with berberine hydrochloride for oral delivery to the inflammation site of ulcerative colitis. AAPS PharmSciTech. 23(6):179. doi: 10.1208/S12249-022-02327-4. 35761150

[CIT0047] Lu Y, Geng W, Li L, Xie F, Zhang M, Xie H, Cai J. 2025. Enhanced antibacterial and antibiofilm activities of quaternized ultra-highly deacetylated chitosan against multidrug-resistant bacteria. Int J Biol Macromol. 298:140052. doi:10.1016/j.ijbiomac.2025.140052. 39832600

[CIT0048] Luo H, Chen M, Song F, Cai X, Yan Y, Li T, Li Y, Songye L. 2024. pH-Sensitive stimulus responsive ZIF-8 composites nanoparticles coated with folic acid-conjugated chitosan for targeted delivery of curcumin. J Clust Sci. 35(5):1533–1547. doi:10.1007/s10876-024-02602-3.

[CIT0049] Mehra M, Sheorain J, Bakshi J, Thakur R, Grewal S, Dhingra D, Kumari S. 2024. Synthesis and evaluation of berberine loaded chitosan nanocarrier for enhanced in-vitro antioxidant and anti-inflammatory potential. Carbohydrate Polymer Technolog Applicat. 7:100474. doi:10.1016/j.carpta.2024.100474.

[CIT0050] Miao Y, Li L, Wang Y, Wang J, Zhou Y, Guo L, Zhao Y, Nie D, Zhang Y, Zhang X, et al. 2024. Regulating protein corona on nanovesicles by glycosylated polyhydroxy polymer modification for efficient drug delivery. Nat Commun. 15(1):1159. doi:10.1038/s41467-024-45254-7. 38326312 PMC10850157

[CIT0051] Mohammadreza R, Ahmad P, Mohammad S, et al. 2023. Effect of curcumin nanoparticles and alcoholic extract of Falcaria vulgaris on the growth rate, biofilm, and gene expression in Pseudomonas aeruginosa isolated from burn wound infection. Mol Biol Rep. 50(8):6681–6690.doi: 10.1007/S11033-023-08559-2. 37378742

[CIT0052] Mondal J, Pillarisetti S, Junnuthula V, Surwase SS, Hwang SR, Park I-K, Lee Y-K. 2023. Extracellular vesicles and exosome-like nanovesicles as pioneering oral drug delivery systems. Front Bioeng Biotechnol. 11:1307878. doi:10.3389/fbioe.2023.1307878. 38260737 PMC10800420

[CIT0053] Monika O, Lucyna H, Przemysław S, et al. 2023. Chitosan nanoparticles-preparation, characterization and their combination with Ginkgo biloba extract in preliminary *in vitro* studies. Molecules. 28(13):4950. doi:10.3390/MOLECULES28134950. PMC10343372 37446611

[CIT0054] Moon AY, Bailey EJ, Polanco JA, Kurata WE, Pierce LM. 2023. Antibacterial efficacy of a chitosan-based hydrogel modified with epsilon-poly-l-lysine against Pseudomonas aeruginosa in a murine-infected burn wound model. Mil Med. 188(Suppl 6):52–60. doi:10.1093/milmed/usad013. 37948238

[CIT0055] Mu D, Zhou L, Shi L, Liu T, Guo Y, Chen H, Luo H, Ma J, Zhang H, Xiong P, et al. 2024. Quercetin-crosslinked chitosan nanoparticles: a potential treatment for allergic rhinitis. Sci Rep. 14(1):4021. doi:10.1038/s41598-024-54501-2. 38369554 PMC10874938

[CIT0056] Muhammad AH, Farah Z, Khalil A, et al. 2023. Synthesis, characterization and evaluation of anti-arthritic and anti-inflammatory potential of curcumin loaded chitosan nanoparticles. Sci Rep. 13(1):10274.doi:10.1038/S41598-023-37152-7. PMC10290721 37355723

[CIT0057] Noura YE, Nadia AM, Nahed AAE, et al. 2023. Evaluation of the in vitro anti-inflammatory and anti-Helicobacter pylori activities of chitosan-based biomaterials modified with copper oxide nanoparticles. Int J Biol Macromol. 2023(P6):253. 10.1016/j.ijbiomac.2023.127277 37806410

[CIT0058] O’La A, Areen A, Rozan OA, et al. 2024. A significant antibiofilm and antimicrobial activity of chitosan-polyacrylic acid nanoparticles against pathogenic bacteria. Saudi Pharmaceutical Journal. 32(1):101918. doi: 10.1016/J.JSPS.2023.101918. PMC10764259 38178849

[CIT0059] Ou K, Wang M, Meng C, Guo K, Shariar Emon N, Li J, Qi K, Dai Y, Wang B. 2024. Enhanced mechanical strength and stretchable ionic conductive hydrogel with double-network structure for wearable strain sensing and energy harvesting. Compos Sci Technol. 255:110732. doi:10.1016/j.compscitech.2024.110732.

[CIT0060] Peng S, Niu S, Gao Q, Song R, Wang Z, Luo Z, Zhang X, Qin X. 2024. Hydroxypropyl chitosan/ε-poly-l-lysine based injectable and self-healing hydrogels with antimicrobial and hemostatic activity for wound repair. [Carbohydr Polym. 337:122135. J doi:10.1016/j.carbpol.2024.122135. 38710549

[CIT0061] Qi Y, Chen Q, Cai X, Liu L, Jiang Y, Zhu X, Huang Z, Wu K, Luo H, Ouyang Q, et al. 2023. Self-assembled amphiphilic chitosan nanomicelles: synthesis, characterization and antibacterial activity. Biomolecules. 13(11):1595. doi:10.3390/biom13111595. 38002276 PMC10669896

[CIT0062] Ray R, Chowdhury SG, Karmakar P. 2024. A vivid outline demonstrating the benefits of exosome-mediated drug delivery in CNS-associated disease environments. Arch Biochem Biophys. 753:109906. doi:10.1016/j.abb.2024.109906. 38272158

[CIT0063] Ren X, Ren J, Li Y, Yuan S, Wang G. 2023. Preparation of caffeic acid grafted chitosan self-assembled micelles to enhance oral bioavailability and antibacterial activity of quercetin. Front Vet Sci. 10:1218025. doi:10.3389/fvets.2023.1218025. 37476826 PMC10354432

[CIT0064] Sally A, Ali MA, Maha N. 2023. Silymarin chitosan-modified penetration enhancer microvesicles as a promising wound healing tool. J Drug Delivery Sci Technol. 2023:84.

[CIT0065] Selin P, Mert P, Aykut Ö, et al. 2023. In vivo evaluation of new adjuvant systems based on combination of Salmonella Typhi porins with particulate systems: liposomes versus polymeric particles. Int J Pharm. 2023:648. 10.1016/j.ijpharm.2023.123568 37925042

[CIT0066] Sepideh A, Tayyebeh M, Mazaher A, et al. 2023. Aerosol assisted synthesis of a pH responsive curcumin anticancer drug nanocarrier using chitosan and alginate natural polymers. Sci Rep. 13(1):19389. doi:10.1038/S41598-023-46904-4. PMC10632444 37938669

[CIT0067] Shahid W, Haroon MA, Maham K, et al. 2024. Green synthesis and antibacterial assessment of chitosan/silver nanocomposite conjugated with tobramycin against antibiotic resistant Pseudomonas aeruginosa. Arabian J Chem. 17(1):105458. doi:10.106/J.ARABJC.2023.105458.

[CIT0068] Shaobing L, Junyu T, Xinfei L, et al. 2022. Baicalin-liposomes loaded polyvinyl alcohol-chitosan electrospinning nanofibrous films: characterization, antibacterial properties and preservation effects on mushrooms. Food Chem. 2022:371. 10.1016/j.foodchem.2021.131372 34808772

[CIT0069] Sharma D, Singh B. 2024. Designing aloe vera-sterculia gum based copolymeric hydrogel dressings for drug delivery. Hybrid Advances. 5:100142. doi:10.1016/j.hybadv.2024.100142.

[CIT0070] Shi J, Hao X, Yang H, He Z, Lu J, Li Y, Luan L, Zhang Q. 2024. A biguanide chitosan-based hydrogel adhesive accelerates the healing of bacterial-infected wounds. Carbohydr Polym. 342:122397. doi:10.1016/j.carbpol.2024.122397. 39048234

[CIT0071] Shi X, Li Y, Kang S, Zhao X, Liu L, Yuan F, He L, Lu H, Liu J. 2024. Dual-functional gallium/chitosan/silk/umbilical cord mesenchymal stem cell exosome sponge scaffold for diabetic wound by angiogenesis and antibacteria. Int J Biol Macromol. 274(Pt 2):133420. doi:10.1016/j.ijbiomac.2024.133420. 38925194

[CIT0072] Shnaikat SG, Shakya AK, Bardaweel SK. 2024. Formulation, development and evaluation of hyaluronic acid-conjugated liposomal nanoparticles loaded with regorafenib and curcumin and their in vitro evaluation on colorectal cancer cell lines. Saudi Pharmaceutical Journal. 32(7):102099. doi:10.1016/j.jsps.2024.102099. 38817822 PMC11135027

[CIT0073] Spogli R, Faffa C, Ambrogi V, D’Alessandro V, Pastori G. 2024. Ozonated sunflower oil embedded within spray-dried chitosan microspheres cross-linked with azelaic acid as a multicomponent solid form for broad-spectrum and long-lasting antimicrobial activity. Pharmaceutics. 16(4):502. doi:10.3390/pharmaceutics16040502. 38675163 PMC11054446

[CIT0074] Srimai C, Sukmongkolwongs W, Manokruang K, Worajittiphon P, Molloy R, Mahomed A, Somsunan R. 2024. Enhancement of poly(vinyl alcohol) hydrogel properties by N-succinyl chitosan and mesona chinensis extract for use as wound dressings. Eur Polym J. 215:113212. doi:10.1016/j.eurpolymj.2024.113212.

[CIT0075] Supapit V, Masubon T, Namfone L, et al. 2023. Ultrasound-assisted formation of chitosan-glucose Maillard reaction products to fabricate nanoparticles with enhanced antioxidant activity. Ultrason Sonochem. 2023:97. 10.1016/j.ultsonch.2023.106466 PMC10267640 37290152

[CIT0076] Tan Y, Xu C, Liu Y, Bai Y, Li X, Wang X. 2024. Sprayable and self-healing chitosan-based hydrogels for promoting healing of infected wound via anti-bacteria, anti-inflammation and angiogenesis. Carbohydr Polym. 337:122147. doi:10.1016/j.carbpol.2024.122147. 38710554

[CIT0077] Tang L, Zhang Z, Ding W, Tang J, Deng X, He Q, Kong X, Chen J, Truong TMH, Wang G, et al. 2024. Preparation, characterization, and Staphylococcus aureus biofilm elimination effect of baicalein-loaded tyrosine/hyaluronic acid/β-cyclodextrin-grafted chitosan nano-delivery system. Int J Biol Macromol. 254(Pt 3):128066. doi:10.1016/j.ijbiomac.2023.128066. 37963503

[CIT0078] Valentino A, Conte R, De Luca I, Di Cristo F, Peluso G, Bosetti M, Calarco A. 2022. Thermo-responsive gel containing hydroxytyrosol-chitosan nanoparticles (Hyt@tgel) counteracts the increase of osteoarthritis biomarkers in human chondrocytes. Antioxidants. 11(6):1210. doi:10.3390/antiox11061210. 35740107 PMC9220116

[CIT0079] Vélez MA, Perotti MC, Zanel P, Hynes ER, Gennaro AM. 2017. Soy PC liposomes as CLA carriers for food applications: preparation and physicochemical characterization. J Food Eng. 212:174–180. doi:10.1016/j.jfoodeng.2017.06.001.

[CIT0080] Wang K, Sun H, Cui Z, Wang J, Hou J, Lu F, Liu Y. 2024. Lactoferrin-chitosan composite hydrogels induced by microbial transglutaminase: potential delivery systems for thermosensitive bioactive substances. J Agric Food Chem. 72(25):14302–14314. doi:10.1021/acs.jafc.4c01551. 38865607

[CIT0081] Wang M, Li Y, Zhao Y, Gao H, Xu Z, Chen L, Liu J, Liang H. 2024. pH-triggered chitosan-sodium caseinate nanocarriers with charge-switching property: characterization and applications in dental care. Food Hydrocolloids. 152:109919. doi:10.1016/j.foodhyd.2024.109919.

[CIT0082] Wang Q, Yan S, Ning Y, Zhu Y, Sergeeva I, Li Y, Qi B. 2024. Effect of sodium alginate block type on the physicochemical properties and curcumin release behavior of quaternized chitosan-oxidized sodium alginate Schiff base hydrogels. Food Chem. 444:138688. doi:10.1016/j.foodchem.2024.138688. 38341919

[CIT0083] Wang T, Cui X, Cai S, Zou X, Zheng S, Li Y, Zhang Z. 2024. Multifunctional phytochemical nanoplatform for comprehensive treatment of all-stage MRSA biofilm associated infection and its accompanying inflammation. Chem Eng J. 480:147951. doi:10.1016/j.cej.2023.147951.

[CIT0084] Wang Y, Wang J, Du H, Zhao Q, Wang S, Liu T, Bi S, Zhang Q, An M, Wen S, et al. 2024. A dynamically cross-linked catechol-grafted chitosan/gelatin hydrogel dressing synergised with photothermal therapy and baicalin reduces wound infection and accelerates wound healing. Int J Biol Macromol. 273(Pt 1):132802. doi:10.1016/j.ijbiomac.2024.132802. 38852721

[CIT0085] Xiaomin Z, Yunfei Z, Li C, et al. 2023. Chitosan-thymol nanoparticle with pH responsiveness as a potential intelligent botanical fungicide against Botrytis cinerea. Pestic Biochem Physiol. 2023:195. 10.1016/j.pestbp.2023.105571 37666600

[CIT0086] Xie Y, Cai P, Xiao H, Pan Y. 2024. Dual-responsive, highly bacteriostatic and swellable chitosan-based hydrogels loaded with 5-fluorouracil for sustainable release. React Funct Polym. 202:105992. doi:10.1016/j.reactfunctpolym.2024.105992.

[CIT0087] Xiong M, Chen Y, Hu H-J, Cheng H, Li W-X, Tang S, Hu X, Lan L-M, Zhang H, Jiang G-B, et al. 2024. Multifunctional pH-responsive hydrogel dressings based on carboxymethyl chitosan: synthesis, characterization fostering the wound healing. Carbohydr Polym. 341:122348. doi:10.1016/j.carbpol.2024.122348. 38876718

[CIT0088] Yu W, You H, Li X, Wang H, Xu J, Chen H, Sun B, Wang X. 2024. pH-responsive chitosan hollow microspheres pro-flavor based on interfacial Schiff-base bonding for controlled release of cinnamaldehyde. Food Hydrocolloids. 156:110260. doi:10.1016/j.foodhyd.2024.110260.

[CIT0089] Yu Y, Kong N, Hou Z, Men L, Yang P, Wang Z. 2024. Sponge-like porous polyvinyl alcohol/chitosan-based hydrogel with integrated cushioning, pH-indicating and antibacterial functions. Int J Biol Macromol. 272(Pt 2):132904. doi:10.1016/j.ijbiomac.2024.132904. 38862323

[CIT0090] Yu Z, Ma L, Ye S, Li G, Zhang M. 2020. Construction of an environmentally friendly octenylsuccinic anhydride modified pH-sensitive chitosan nanoparticle drug delivery system to alleviate inflammation and oxidative stress. Carbohydr Polym. 236(prepublish):115972. doi:10.1016/j.carbpol.2020.115972. 32172827

[CIT0091] Yuan X, Wei Y, Villasante A, Ng JJD, Arkonac DE, Chao P-HG, Vunjak-Novakovic G. 2017. Stem cell delivery in tissue-specific hydrogel enabled meniscal repair in an orthotopic rat model. Biomaterials. 132:59–71. doi:10.1016/j.biomaterials.2017.04.004. 28407495 PMC5473162

[CIT0092] Yuan Y, Chen Q, Wang Z, Mi Y, Dong F, Tan W, Guo Z. 2024. Low molecular weight chitosan based GSH-responsive self-assembled cationic micelle with enhanced anti-tumor effect by combining oxidative damage and chemotherapy. Int J Biol Macromol. 268(Pt 2):131736. doi:10.1016/j.ijbiomac.2024.131736. 38653433

[CIT0093] Zhang L, Shen B, Zheng C, Huang Y, Liang Y, Fei P, Chen J, Lai W. 2024. Chitosan/oxidized sodium alginate/Ca2+ hydrogels: synthesis, characterization and adsorption properties. Food Hydrocolloids. 156:110368. doi:10.1016/j.foodhyd.2024.110368.

[CIT0094] Zhang W, Zhang Q, Yang Y, Chen Y, Wei J, Lu F, Li D. 2025. Multi-functional chitosan polymeric micelles for improving the oral bioavailability of paclitaxel based on synergistic effect. Drug Deliv Transl Res. 15(1):312–324. doi:10.1007/s13346-024-01597-8. 38643258

[CIT0095] Zhang X, Lai Y, Zhang L, Chen Z, Zhao J, Wang S, Li Z. 2024. Chitosan-modified molybdenum selenide mediated efficient killing of Helicobacter pylori and treatment of gastric cancer. Int J Biol Macromol. 275(Pt 1):133599. doi:10.1016/j.ijbiomac.2024.133599. 38960263

[CIT0096] Zhang Z, Ge M, Wu D, Li W, Chen W, Liu P, Zhang H, Yang Y. 2024. Resveratrol-loaded sulfated Hericium erinaceus β-glucan-chitosan nanoparticles: preparation, characterization and synergistic anti-inflammatory effects. Carbohydr Polym. 332:121916. doi:10.1016/j.carbpol.2024.121916. 38431417

[CIT0097] Zhong C, Zou J, Mao W, Yang P, Zhang J, Gou S, Zhang Y, Liu H, Ni J. 2024. Structure modification of anoplin for fighting resistant bacteria. Eur J Med Chem. 268:116276. doi:10.1016/j.ejmech.2024.116276. 38452726

[CIT0098] Zhu L, Ouyang F, Fu X, Wang Y, Li T, Wen M, Zha G, Yang X. 2024. Tannic acid modified keratin/sodium alginate/carboxymethyl chitosan biocomposite hydrogels with good mechanical properties and swelling behavior. [Sci Rep. 14(1):12864. J doi:10.1038/s41598-024-63186-6. 38834664 PMC11150462

